# Amelioration effect of 18β-Glycyrrhetinic acid on methylation inhibitors in hepatocarcinogenesis -induced by diethylnitrosamine

**DOI:** 10.3389/fimmu.2023.1206990

**Published:** 2024-01-15

**Authors:** Hany Khalil, Alaa H. Nada, Hoda Mahrous, Amr Hassan, Patricia Rijo, Ibrahim A. Ibrahim, Dalia D. Mohamed, Fawziah A. AL-Salmi, Doaa D. Mohamed, Ahmed I. Abd Elmaksoud

**Affiliations:** ^1^ Department of Molecular Biology, Genetic Engineering and Biotechnology Research Institute (GEBRI) University of Sadat City, Sadat, Egypt; ^2^ Department of Industrial Biotechnology, Genetic Engineering and Biotechnology Research Institute (GEBRI) University of Sadat City, Sadat, Egypt; ^3^ Department of Bioinformatics, Genetic Engineering and Biotechnology Research Institute (GEBRI) University of Sadat City, Sadat, Egypt; ^4^ Research Center for Biosciences & Health Technologies (CBIOS), Universidade Lusófona de Humanidades e Tecnologias, Lisboa, Portugal; ^5^ Instituto de Investigação do Medicamento (iMed.ULisboa), Faculdade de Farmácia, Universidade de Lisboa, Lisboa, Portugal; ^6^ Department of Plant Biotechnology, Genetic Engineering and Biotechnology Research Institute (GEBRI) University of Sadat City, Sadat, Egypt; ^7^ Department of Biology, Faculty of Sciences, Taif University, Taif, Saudi Arabia; ^8^ College of Biotechnology, Misr University of Science and Technology, Giza, Egypt

**Keywords:** 18β-Glycyrrhetinic acid (GA), hepatocellular carcinoma (HCC), *lactobacillus rhamanosus*, DLC-1 and TET-1, NF-kB, STAT-3 methylation inhibitors, epigenetics

## Abstract

**Aim:**

suppression of methylation inhibitors (epigenetic genes) in hepatocarcinogenesis induced by diethylnitrosamine using glycyrrhetinic acid.

**Method:**

In the current work, we investigated the effect of sole GA combined with different agents such as doxorubicin (DOX) or probiotic bacteria (*Lactobacillus rhamanosus*) against hepatocarcinogenesis induced by diethylnitrosamine to improve efficiency. The genomic DNA was isolated from rats’ liver tissues to evaluate either methylation-sensitive or methylation-dependent resection enzymes. The methylation activity of the targeting genes DLC-1, TET-1, NF-kB, and STAT-3 was examined using specific primers and cleaved DNA products. Furthermore, flow cytometry was used to determine the protein expression profiles of DLC-1 and TET-1 in treated rats’ liver tissue.

**Results:**

Our results demonstrated the activity of GA to reduce the methylation activity in TET-1 and DLC-1 by 33.6% and 78%, respectively. As compared with the positive control. Furthermore, the association of GA with DOX avoided the methylation activity by 88% and 91% for TET-1 and DLC-1, respectively, as compared with the positive control. Similarly, the combined use of GA with probiotics suppressed the methylation activity in the TET-1 and DLC-1 genes by 75% and 81% for TET-1 and DLC-1, respectively. Also, GA and its combination with bacteria attenuated the adverse effect in hepatocarcinogenesis rats by altering potential methylomic genes such as NF-kb and STAT3 genes by 76% and 83%, respectively.

**Conclusion:**

GA has an ameliorative effect against methylation inhibitors in hepatocellular carcinoma (HCC) by decreasing the methylation activity genes.

## Introduction

Hepatocarcinogenesis is one of the most aggressive common cancers in the world (1). The majority of primary liver cancers (80%) are HCC, followed by cholangiocarcinomas (10–20%) and angiosarcomas (1%) (2). Hepatocellular carcinoma (HCC) is the ninth most frequent cancer in women and the fifth most frequent in men (3). Hepatitis B and C virus (HBV and HCV), alcoholic fatty liver disease (AFLD), and non-alcoholic fatty liver disease (NAFLD) are major risk factors leading to an increase in the incidence of HCC (4). many strategies to induce HCC in mice, such as genetically engineered mice, chemotoxic agents, implantation models, or humanized mice (5). The chemotoxic agent method is utilized in several mouse models to induce tumor formation. Induction of HCC tumorigenesis by a chemotoxic agent occurs through two paths: initial tumor formation by a hepatotoxic compound that promotes clonal expansion of preneoplastic cells or direct DNA damage (6). Diethylnitrosamine (DEN) is a carcinogen that has been used to induce HCC in mice for decades. The ability of DEN to transfer into alkylate DNA and generate oxidative stress by indicating reactive oxygen species production leading to DNA damage (7) 18β-Glycyrrhetinic acid (GA) is extracted from traditional Chinese medicine licorice (Glycyrrhiza glabra) by hydrolyzing the glycyrrhizin product with a pentacyclic triterpene glycoside (8). Previous reports mentioned that GA was used for the treatment of liver disease in Aisa due to its hepatoprotective activity (9). Furthermore, past reports demonstrated that GA is a good anticancer agent against HCC by different mechanisms, including activation of autophagy and apoptosis (10), cycle arrest (11), or reduction of immunosuppression (12). There is a relationship between immunotherapy response rates and the gut microbiome community structure. Previous studies reported the influence of *Lactobacillus rhamnosus* (LGG) to induce an anti-inflammatory response in the intestine by regulating interleukin (IL)10 levels and promoting regulatory T-cell activity (13). Understanding hypomethylation and hypermethylation processes can help understand various diseases, including cancer, and potential treatments. This is because epigenetics significantly influences how cells function (14). DNA-methyltransferase enzymes (DNMTs) catalyze DNA methylation. There are different enzymes, such as DNMT1, which controls the DNA methylation pattern, and DNMT2, which catalyzes RNA methylation. The negative methylation was maintained by DNMT3a and DNMT3b (15). Methylation and demethylation are in equilibrium. Demethylation reactions are catalyzed by DNA methylase. Within CpG islands, DNMTs preferentially methylate cytosine. CpG islands are CG-rich DNA regions upstream of genes containing gene promoters and controlling gene expression through varying methylation levels. About 40% of all gene promoters are thought to contain CpG islands, and almost half of all CpG islands are found in housekeeping genes (16). CpG islands are domains that recruit RNA polymerase II and transcription factors to begin transcription (17). CpG methylation promotes the binding of methyl-binding proteins, resulting in nucleosome condensation and, as a result, transcription inhibition (18). The ten-eleven translocation 1 (TET1) plays a central role in the demethylation process via catalyzation, where an active hydroxylates from 5mC to 5hmC *in vivo* and *in vitro* (19). There are three mechanisms for active demethylation by TET1: TET1 protein prevents DNA from maintenance methylation, TET1-mediated active demethylation in DNA repair, or TET-1-mediated decarboxylation of 5caC (20). Deleted in liver cancer 1 (DLC-1) has a great active role in inactive tumor suppressor gene deletion in hepatic cancer because it is induced by promoter methylation (21). Recently, scientists suggested that DLC-1 plays a critical role in tumorigenesis in several cancer models, such as gallbladder carcinoma (22), gastric cancer (23), and hepatocellular carcinoma (24). Also, DLC-1 inactivation is induced by methylation (25). STAT3 is only transiently activated in the liver under physiological conditions due to the tight control of downregulation. The IL-6/STAT3 pathway in hepatocytes is involved in hepatoprotection after liver damage (26) and glucose homeostasis by inhibiting gluconeogenesis in response to an increase in plasma insulin (27). It has been well documented that STAT3 plays an oncogenic role in HCC, stimulating growth, anti-apoptosis, migration, invasion, angiogenesis, cancer stem cell properties, and immune suppression of cancer cells (14). These actions are generally carried out by controlling the transcription of several oncogenic target genes. Chronic inflammation and the subsequent production of proinflammatory cytokines can activate the NF-kb signaling pathway, potentially harmful to the liver (28). Cooperation between STAT3 and NF-kB may also occur, given that approximately one-third of HCC tumors activate both STAT3 and NF-kB (29). Furthermore, STAT3-mediated microRNA (miRNA) expression is emerging as an epigenetic mechanism for driving hepatic oncogenesis, and miRNA can also play a role in STAT3 signaling regulations (30, 31). We sought to investigate the methylomic change in tumor suppressor genes (*TET-1* and *DLC-1*) and oncogenes represented by NF-kb and STAT-3 and subsequently confirm our data with the relative gene expression profile of all indicated genes upon treatment with GA, a combination of GA with DOX, and GA with bacteria (*lactobacillus rhamanosus*).

## Materials and methods

### Induction of hepatocarcinogenesis

According to the Institutional Animal Care and Use Committee at the Faculty of Veterinary Medicine, University of Sadat City, Sadat City, Egypt (Ethical approval number: VUSC-025-1-22). We carried out the experimental procedure after the Animal Protocols Evaluation Committee’s affirmative opinion. Twenty-four male albino rats (180–200 g) of the Wistar strain were used. All animals in the study were housed in pathogen-free facilities under a 12-hour light/dark cycle at constant temperature and humidity and were fed standard rodent chow and water ad libitum (1).

For studies of liver tumor development, 15-day-old rats were treated with a single dose of DEN (Sigma-Aldrich) given dissolved in saline at a dose of 25 mg/kg body weight by i.p. injection. Rats in one randomly pre-assigned group were killed 4 months after DEN administration for histological and biochemical analyses (32).

### Animals and experimental design

In the current study, rats were divided into seven groups arranged as follows:

Group 1: Negative control animal.
Group2: positive control animal (rats with HCC).
Group 3: animal administrated received GA (100 mg/kg. orally) daily for 4 weeks.
Group 4: Rats were injected with Doxorubicin (50 mg/kg) daily by i.p for 4 weeks.
Group 5: The rats treated with probiotic bacteria (*Lactobacillus rahmnosus*) (2x10^8^ CFU/mL) were given it daily for the 4 weeks.
Group 6: The rats was oral administered probiotic bacteria (2x 10^8^ CFU/mL) with GA (100 mg/kg) daily for the 4 weeks.
Group 7: Oral administration of Dox (50 mg/kg) with GA (100 mg/kg) daily for 4 weeks.

### Histopathological examination

After the experimental period, rats fasted overnight, and then blood samples were taken from the heart under diethyl ether anesthesia. Rats were sacrificed by decapitation; the whole liver was dissected, and a small part from the right lobe of the liver was cut and fixed in 10% phosphate-buffered formalin (pH 7.2) for histopathological examination. The remaining liver was stored at -80°C. Liver tissue samples were fixed in 10% neutral buffered formalin. The fixed specimens were then trimmed, washed, and dehydrated in escalating grades of alcohol, cleared in xylene, embedded in paraffin, sectioned at 4-6 U thickness, and stained with hematoxylin and eosin.

### Sample preparation

The liver tissues of sacrificed mice were crushed in liquid nitrogen using phosphate-buffered saline (PBS) supplemented with 1% fetal bovine serum (FBS). Then the crashed tissues were homogenized, filtered using a 0.25µm filter, and collected in RNase- and DNase-free Eppendorf tubes. The homogenized and purified cells were used for total RNA isolation and flow cytometry staining.

### DNA isolation and methylation activity

The genomic DNA of liver tissue from the prepared rats was isolated using a DNA purification kit (Qiagen) according to the manufacturing protocol. To investigate the methylation activity in the coding sequences of DLC-1, TET-1, NF-kB, and STAT-3 genes, genomic DNA was digested for 4 hours at 37°C with either methylation-sensitive or methylation-dependent resection enzymes provided in the EpiTect Methyl II DNA Restriction Kit (Qiagen, USA). Quantitative real-time PCR (qRT-PCR) was used to achieve the methylation activity in the coding sequences of the indicated genes using the specific primers listed in **Table 1** and cleaved DNA products. The following amplification program was used: 95°C for 10 min, 40 cycles (95°C for 45 sec, 62°C for 30 sec, and 72°C for 45 sec), and finally 72°C for 10 min. The mean cycle threshold (Ct) outcome using cleaved DNA by methylation-sensitive restriction was considered a methylated level, while the mean Ct value using cleaved DNA by methylation-dependent cleaved DNA was considered an unmethylated level. The methylation activities were calculated according to delta-delta Ct (ΔΔct) equations. Delta Ct (Δct) was equal to Ct for the methylated value and Ct for the unmethylated value. ΔΔct was equal to Δct for the sample and Δct for the control. Finally, the methylation fold change was equivalent to 2-ΔΔct (33).

**Table 1:** Oligonucleotides sequences used for mRNA quantification of indicated genes.

Description	Primer sequences 5’-3’
**DNMT-1 sense**	AGGAATGTGTGAAGGAGAAATTG
**DNMT-1 antisense**	CTTGAACGCTTAGCCTCTCCATC
**MS sense**	AGAAGAGGATTATGGTGCTGGATG
**MS antisense**	TCTTAATTCCTGTCTGGAGAGTT
**TET1- sense**	ACTCCCTGAGGTCTGTCCTGGGA
**TET1- antisense**	GGATCGAGACATAGCTACAGAGT
**DLC-1-sense**	CCGCCTGAGCATCTACG
**DLC-1-antisense**	ACTATCCGCTGCATCCC
**NF-kB1-antisense**	ATCACTTCAATGGCCTCTGTGTAG
**NF-kB1-antisense**	ATAGGCACTGTCTTCTTTCACCTC
**STAT-3-sense**	ACCCAACAGCCGCCGTAG
**STAT-3-antisense**	CAGACTGGTTGTTTCCATTCAGAT
**GAPDH-sense**	TGGCATTGTGGAAGGGCTCA
**GAPDH-antisense**	TGGATGCAGGGATGATGTTCT

### Sodium bisulfite DNA-converted protocol

According to the manufacturer’s protocol, the genomic DNA was isolated from the obtained samples using a DNA purification kit (Qiagen, USA). Bisulfite analysis of the DLC-1 and TET-1 promoter regions was performed using the spin columns of the EpiTect Bisulfite Kit (Qiagen, USA). According to the manufacturer’s protocol, the purified genomic DNA (1 µg) was treated with 100 µl sodium bisulfite (10M) and 100 µl sodium chloride (1M). The reaction was thermally denaturized for 5 minutes at 95°C and incubated for 6 hours at 55°C (3 cycles) for complete conversion of unmethylated cytosine. The converted DNA was then applied to PCR amplification using the following unmethylated and methylated specific primers, as displayed for DLC-1 and TET-1 promoter regions; DLC1-Unmethylated-F-5- AAACCCAACAAAAAAACCCAACTAACA- 3, DLC1- Unmethylated -R-5- TTT TTTAAA GAT TGAAATGAGGGAGTG -3, DLC1-Methylated-F-5- CCCAACGAA AAAACC CGACTAACG-3, DLC1- Methylated-R-5-TTTAAAGATCGAAACGAGGGAGCG -3, TET1-Unmethylated-F-5- GGCTCGGGCCTTGACTGTGCTG -3, TET-Unmethylated-R-5-AGGTTTTGGTCGCTGGCCGGGT -3, TET1-Methylated-F-5-AACTCAAACCTTAACTATACTA -3, TET1-Methylated-R-5- CGCTAACCGAATCACATTCCCA-3 (27). The conventional PCR was used to amplify the methylated and unmethylated fragments in the bisulfite-converted DNA using the following parameters: 95 °C for 5 min and 40 cycles (95 °C for 45 sec, 62 °C for 45 sec, and 72 °C for 45 sec) (23). The PCR products were loaded in 1% agarose gel and electrophoresed using a 1X-TBE buffer, and the amplified fragments were monitored using the UV trans-illuminator with a long-wave (320 nm) UV and gel documentation system.

### Flow cytometry analysis

Flow cytometry was used to evaluate the protein expression profiles of DNMT1, MS, DLC-1, TET-1, NFkB, and STAT3 in the liver tissue of treated rats. Accordingly, drug-treated homogenized cells were centrifuged for 3 minutes at 1500 rpm. The supernatant was discarded; the pellet was resuspended in PBS for washing and then centrifuged and resuspended in cold methanol for fixation. The cells were resuspended in PBS for permeabilization, including Triton-X-100 (0.1%), and incubated for 3 min. For staining of DLC-1, the cells were resuspended and incubated overnight at 4°C in PBS supplemented with 1% BSA and diluted mouse monoclonal anti-DLC-1 (Sigma-Aldrich, Germany). After washing, the cells were centrifuged and resuspended in PBS that contains 1% BSA and 1-1000 secondary antibody donkey anti-mouse IgG (Alexa Fluor 488, Invitrogen, Germany). The same conditions were followed in the staining of TET-1 protein in treated cells using rabbit polyclonal anti-TET-1 (PromoCell, Germany) and goat anti-rabbit IgG (Alexa Fluor 594, Abcam, USA). The same procedures were considered for staining DNMT1, MS, NFkB, and STAT3 using specific antibodies: mouse monoclonal anti-DNMT1 (Sigma-Aldrich, Germany), rabbit monoclonal anti-MS (Sigma-Aldrich, Germany), mouse monoclonal anti-NFkB (Sigma-Aldrich, Germany), and rabbit monoclonal anti-STAT3 (Sigma-Aldrich, Germany), respectively. The same secondary antibodies, donkey anti-mouse IgG (Alexa Fluor 488, Invitrogen, Germany) and goat anti-rabbit IgG (Alexa Fluor 594, Abcam, USA), were used independently to achieve the kinetic protein expression of each target. Finally, the flow cytometry assay (BD Accuri 6 Plus) was used to assess the protein levels using a resuspended pellet in 500 µl PBS (34, 35).

### RNA isolation and qRT-PCR

Total RNA was isolated from rat-prepared tissues using TriZol (Invitrogen, USA), chloroform, and isopropanol. Then the total RNA was purified by using an RNA purification kit (Invitrogen, USA). According to the manufacturer’s protocol, the complementary DNA (cDNA) was prepared from total RNA using the QuantiTech Reverse Transcriptase Kit (Qiagen, USA). The relative gene expression of DNMT-1, MS, and the mRNA expression of the indicated genes was quantified using the QuantiTect SYBR Green PCR Kit (Qiagen, USA). Ct values of the housekeeping gene, GAPDH, were used for normalization. The following PCR parameters were used to assess fold changes in the indicated gene expression: 94°C for 5 min, 40 cycles (94°C for 30 sec, 60°C for 30 sec, 72°C for 45 sec). Delta-delta Ct equations have been used to determine the relative gene expression indicated by fold changes in quantified mRNA (36–38).

### Statistical analysis

The data obtained by q-RT-PCR reveal that the cycle threshold values (Ct) were analyzed using the previously described ΔΔCt equations. Hence, the relative gene expression of targeted genes on the RNA level was indicated by fold changes that equaled 2-ΔΔct (23, 33, 34). The student’s two-tailed t-test was used to determine the differences in the analyzed data. P<0.05 was considered statistically significant (*), while P<0.01 was considered highly significant (**).

## Results

### The effect of GA on the body weight and liver weight

**Figure 1:**
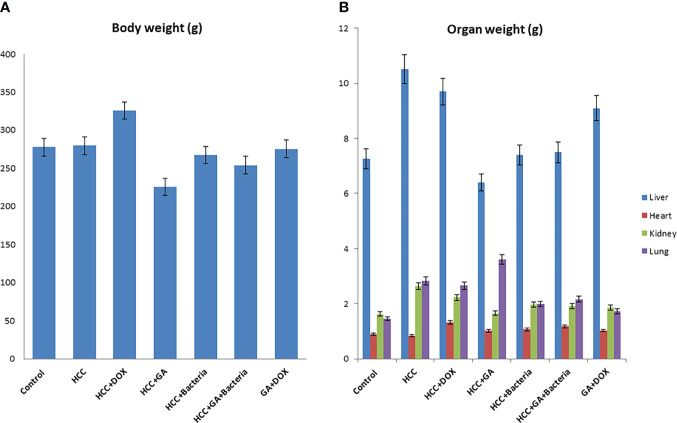
Chemical induction of HCC in albino rats treated with different effectors. **(A)** Body weight after treatment of HCC with different effectors. **(B)** Organ weight (lung, heart, kidney and liver) after treatment of HCC with different effectors.

Hepatocarcinogenesis of the rats was induced by using DEN; then, the animals were treated with different agents such as GA, DOX, an association of GA with DOX, and a combination of GA and probiotics. As shown in **Figure 1**, GA decreases body weight by 25% as compared to positive controls (HCC animals). While the DOX drug contributes to increasing the body weight of the animal by 20% as compared to the positive control, **Figure 1B** displays the effect of the different agents on the organ’s weight. GA has a significant effect on decreasing the liver weight by 33.3% as compared to positive control animals (HCC animals), while GA increases the weight of the lung by 22% as compared to positive control animals. Also, the GA has a significant effect on reducing the kidney weight by 16% as compared to the positive control (HCC animals). There is no significant difference in the heart weight of all the groups. The **Supplementary Figure 1** indicated the effect of the association of GA with probiotics and showed normal liver size and color compared with untreated rats with HCC.

### The effect of GA on Histopathological of rats’ liver

**Figure 2:**
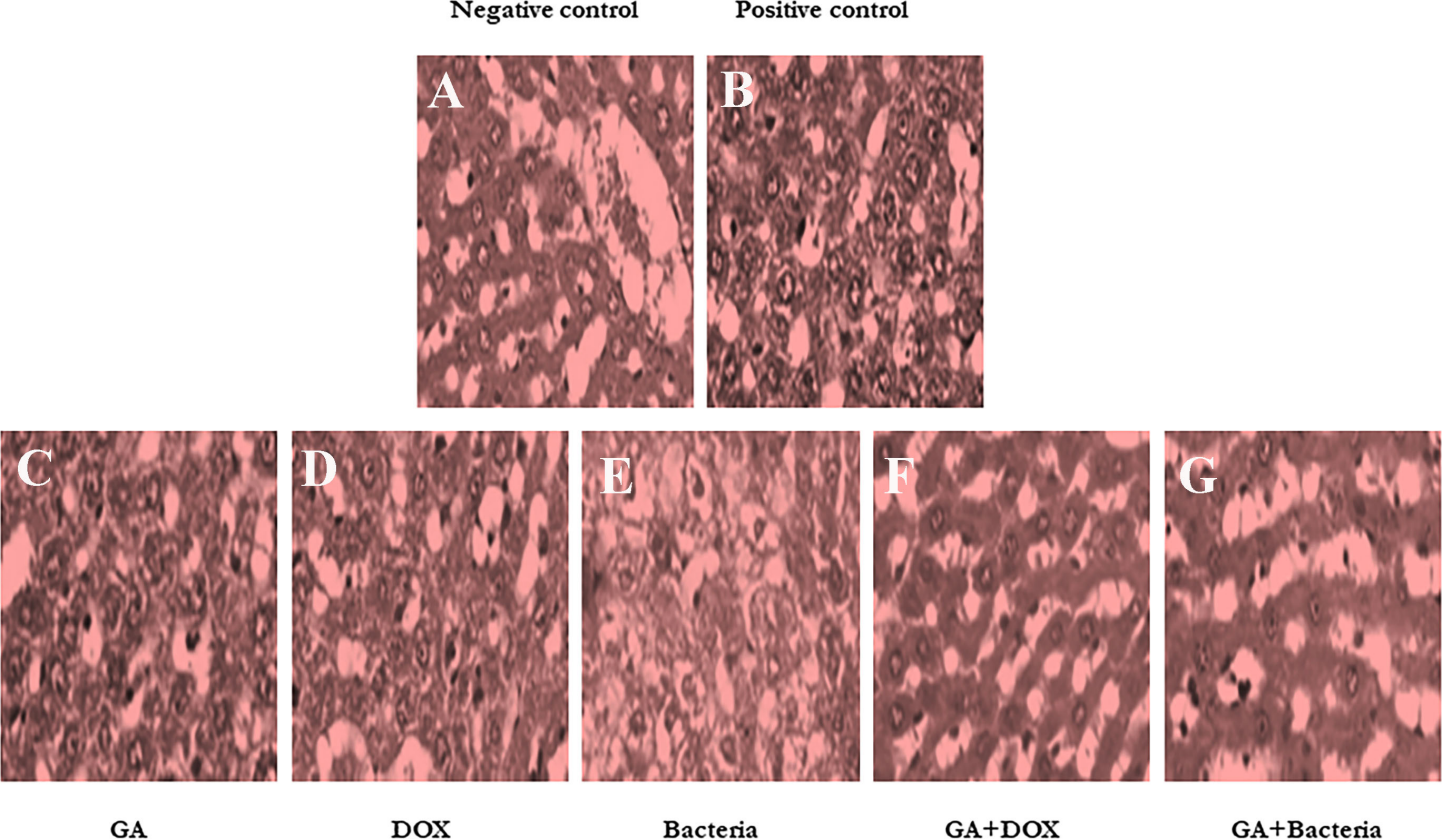
Histological studies on evaluation of different compounds against HCC induced in albino rats A photomicrographic section of normal liver from the negative control group clearly shows normal liver morphology, whereas the positive control carcinogenic group demonstrates necrobiotic alterations and focal pleomorphic neoplastic cells, and rat liver treated with DOX illustrates mild swelling of hepatic carcinoma cells with hyperplasia of Kupffer cells. Poor <50% tumor necrosis, but with rat liver treated with GA showing necrobiotic changes of hepatic carcinoma cells with 51%-99% tumor necrosis, while also rat liver treated with GA and DOX showing small focal necrotic areas with apoptosis of a few hepatocytes, rat liver treated with bacteria showing vacuolar degeneration of hepatic carcinoma cells with hyperplasia of Kupffer cells and poor <50%percent tumor necrosis, and photomicrograph of rat liver section demonstrated necrobiotic changes of hepatic carcinoma cells. **(A)** Negative control animal. **(B)** positive control animals (rats with HCC). **(C)** The animal administrated received GA (100 mg/kg. orally) daily for 4 weeks. **(D)** Rats were injected with Doxorubicin (50 mg/kg) daily by i.p for 4 weeks. **(E)** The rats treated with probiotic bacteria (Lactobacillus rahmnosus) (2x108 CFU/mL) were given it daily for 4 weeks. **(F)** The rats treated with Dox (50 mg/kg) with GA (100 mg/kg) daily for 4 weeks. **(G)** The rats were treated with probiotic bacteria (2x 108 CFU/mL) with GA (100 mg/kg) daily for 4 weeks.

**Figure 2** shows that the negative control animals (normal animals) group showed normal-textured tissues of hepatic lobules with regular hepatic cords and a prominent central hepatic vein. The hepatic cells were joined in the organizing plate’s grade (0) (**Figure 2A**). The hepatic carcinogenic group (positive control) showed neoplastic areas with moderately differentiated grade II large hepatic cells with deeply basophilic cytoplasm and hyperchromatic nuclei, which indicated malignancy in the form of cellular and nuclear pleomorphism, scanty cytoplasm, and frequent mitotic The hepatic lobule displayed disorganization of hepatic cords and focal areas of necrosis and fatty change with hyperplasia of Kupffer cells. **Figure 2B**. The carcinogenic animal group treated by GA showed necrobiotic changes in hepatic carcinoma cells with hyperplasia of Kupffer cells. A partial 51%–99% of tumor necrosis was seen. The necrotic areas showed nuclear pyknosis and deeply eosinophilic cytoplasm. Disorganization of carcinoma cells with widening of hepatic sinusoids was noticed in **Figure 2C**. The hepatocarcinogenesis animal group treated with DOX showed a mild swelling of hepatic carcinoma cells with hyperplasia of Kupffer cells. Less than 50% of tumor necrosis was seen. There are apoptotic cells in the small focal necrotic areas in a small number of hepatocytes (**Figure 2D**). The liver tissue section of the carcinogenic group treated by bacteria showed vacuolar degeneration of hepatic carcinoma cells with hyperplasia of Kupffer cells. Poor (<50%) tumor necrosis was seen. The necrotic cells showed nuclear pyknosis and deeply eosinophilic cytoplasm (**Figure 2E**). The hepatocarcinogenesis animal group treated with a combination of DOX and GA showed mild swelling of hepatic carcinoma cells with hyperplasia of Kupffer cells. Partial (51%–99%) tumor necrosis was seen. The small focal necrotic areas showed apoptosis of a small number of hepatocytes (**Figure 2F**). The hepatocarcinogenesis animal group treated with GA associated with probiotics showed necrobiotic changes in hepatic carcinoma cells with hyperplasia of Kupffer cells. Partial 51%–99% tumor necrosis was seen. The necrotic areas showed nuclear pyknosis and deeply eosinophilic cytoplasm. The disorganization of carcinoma cells with the widening of hepatic sinusoids was noticed in **Figure 2G**. Finally, the cell morphology of HCC-developed rates treated with GA, a combination of GA with bacteria, and GA with Dox showed regular statements of liver cells. This observation suggests that treatment with the indicated compounds can restore liver cancer cells.

### DNMT1 and MS markedly depleted in rates with HCC upon treatment with GA, probiotic bacteria, and their combinations

**Figure 3:**
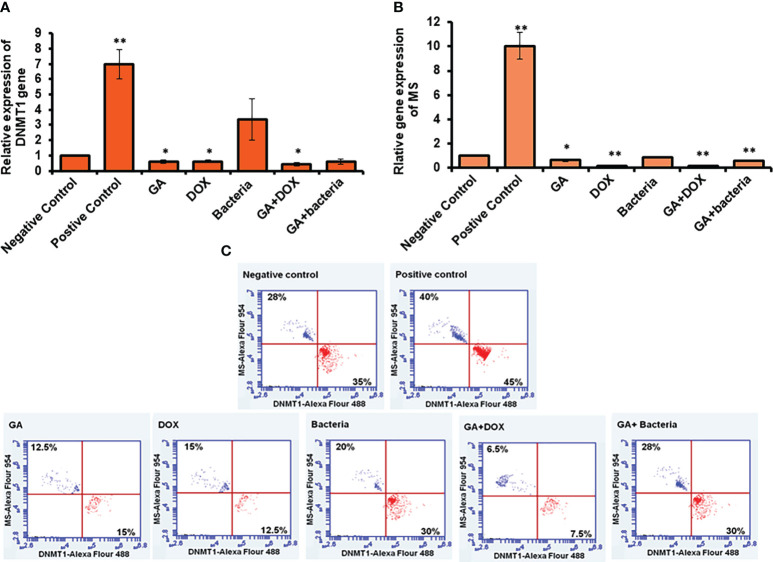
Measurement of the cofactor of methylation activity (DNMT1and MS) by qRT-PCR and flow cytometery. Positive control increased DNMT1 to 7-fold change while other treatment makes significant downregulation **(A)**. Error bars indicate standard deviation of three independent experiments. The student’s two-tailed t-test was used for statistical analysis. P-value ≤ 0.05 was considered statistically significant. Positive control increased MS to 10-fold change while other treatment makes significant downregulation **(B)**. Protein expression profile of DNMT1 and MS indicated by red and blue dotes using secondary antibodies Alex Flour-488 and 594, respectively **(C)**. *statistically significant difference as compared with the controls (P, 0.05 for each). While, ** statistically significant difference as compared with the controls (P, 0.01 for each).

To investigate the possible involvement of DNA methylation in restoring liver cancer cells, the relative gene expression of both DNMT1, as the enzyme responsible for DNA methyl ion activity and methionine synthase (MS), as the enzyme facilities performing methyl groups was detected in liver tissue. Interestingly, the relative gene expression of DNMT1 significantly increased in rates with well-differentiated HCC (positive control). Meanwhile, its relative gene expression strongly downregulated in rats treated with the GA, a combination of GA with bacteria, and GA with Dox (**Figure 3A**). The relative gene expression of MS increased in positive control rats. While, its expression was significantly reduced in rats treated with GA, a combination of GA with bacteria, and GA with Dox GA. Notably, MS gene expression showed neglected differentiation in treated rates with probiotic bacteria compared with other treated rats (**Figure 3B**). Interestingly, the protein expression profile of DNMT1 and MS was markedly increased in rats with HCC (positive control) up to 45% and 40% of stained cells compared with the negative control rats indicated by flow cytometry (**Figure 3C**). In addition, their expression profile was notably reduced to a percentage of about 15% of stained cells in rats treated with GA or Dox. While combining GA and Dox during treatment, the rats successfully reduced the expression of DNMT1 and MS protein to about 7% of stained cells (**Figure 3C**). This result demonstrates the hypermethylation activity in HCC-developed rats indicated by DNMT1 and MS expression profile which have been reduced upon treatment with the GA, combination of GA with Dox.

### Methylation changes in *TET-1* and *DLC-1* promoter region in response to HCC development and GA treatment

**Figure 4:**
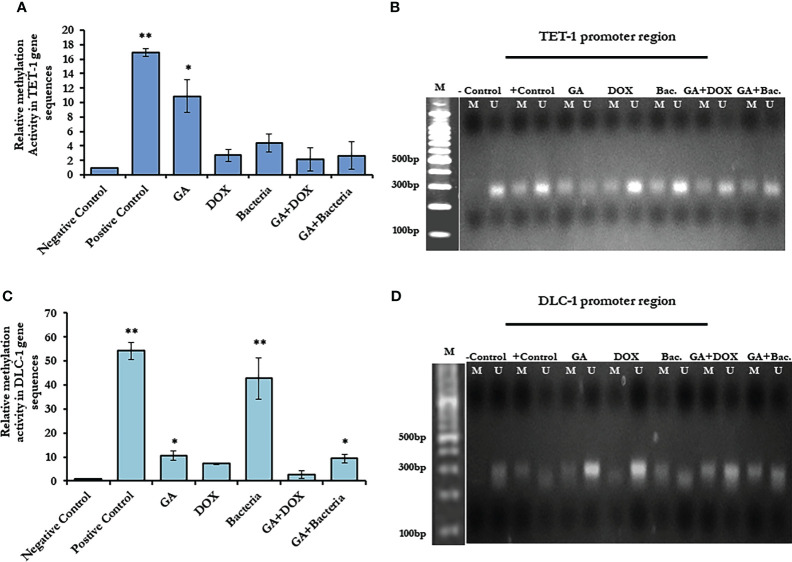
Methylation activity based on relative methylation in TET1 and DLC-1 in coding sequence and by restriction enzyme on the promoter region. **(A)** Fold change of methylated activity of TET-1 based on coding sequence. **(B)** Agarose gel electrophoresis of TET-1 gene segment that digested with CpG restriction enzyme HpaII indicates all derived samples in rats treated with GA, DOX, a combination of GA with bacteria and combination of GA with DOX in addition to controlling samples. **(C)** The methylation activity of DLC-1 is indicated by methylation fold change. The error bar indicates the stander deviation between two different replicates; statically, the student’s two-tails t-test test has been differentiating significantly. **(D)** Agarose gel electrophoresis of DLC-1 gene segment that digested with CpG restriction enzyme HpaII indicated all derived samples in rats treated with GA, DOX, a combination of GA with bacteria, and a combination of GA with DOX in addition to control samples. *statistically significant difference as compared with the controls (P, 0.05 for each). While, ** statistically significant difference as compared with the controls (P, 0.01 for each).

To investigate the possible epigenetic alteration in the coding sequence of *TET-1* and *DLC-1* genes, methylation-dependent and methylation-sensitive enzymes have been used to digest the purified genes using qRT-PCR. Interestingly, the methylation activity in *TET1* increased up to a 16-fold change in rats with well-differentiated HCC, while it increased up to a 50-fold change in *DLC1* as presented in (**Figure 4A**; **Supplementary Table 1**). Notably, GA treatment sufficiently decreased the methylation activity in both genes to only a 10-fold change. Furthermore, the combination of GA with DOX or bacteria successfully avoids the methylation activity in both genes (**Figures 4A, C**; **Supplementary Table 1**). Based on the principle of sodium bisulfate treatment indicated that all unmethylated cytosine is transferred to uracil after treatment with sodium bisulfate (39). Thus, the methylated and unmethylated primers that could be complementary with converted and non-converted cytosine were used to detect the unmethylated and methylated fragments, respectively. According to the gel electrophoresis presented in (**Figures 4B, D**), the methylated band of *TET-1* and *DLC-1* disappeared in the negative control while showing the detectable band in the positive control. Interestingly, GA treatment showed a faint band of methylated and unmethylated TET1 promoter while showing a strong band of unmethylated DLC1 promoter, indicating the role of GA in decreasing the methylation activity in the promoter region of both *TET1* and *DLC1*. The combination of GA and DOX showed a faint band of the methylated and unmethylated promoter region of *DLC1* compared to the promoter region of *TET1*. The combination of GA with the bacteria determined a faint band of the methylated promoter region of both *TET1* and *DLC1*. Together, these data indicate the anti-methylation activities of GA, in addition to DOX during HCC treatment.

### Restoring of TET1 and DLC1 expression profile in rats with HCC in response to GA treatment

To investigate the possible involvement of DNA methylation in restoring liver cancer cells, the relative gene expression of both DNMT1, the enzyme responsible for DNA methyl ion activity, and methionine synthase (MS), the enzyme facility performing methyl groups, was detected in liver tissue. Interestingly, the relative gene expression of DNMT1 significantly increased in rates with well-differentiated HCC (positive control). Meanwhile, its relative gene expression was strongly downregulated in rats treated with GA, a combination of GA with bacteria, and GA with Dox (**Figure 3A**). The relative gene expression of MS increased in positive control rats. While its expression was significantly reduced in rats treated with GA, a combination of GA with bacteria, and GA with Dox GA, Notably, MS gene expression showed neglected differentiation in treated rates with probiotic bacteria compared with other treated rats (**Figure 3B**). Interestingly, the protein expression profile of DNMT1 and MS was markedly increased in rats with HCC (positive control) up to 45% and 40% of stained cells, respectively, compared with the negative control rats, as indicated by flow cytometry (**Figure 3C**). In addition, their expression profile was notably reduced to a percentage of about 15% of stained cells in rats treated with GA or Dox. While combining GA and Dox during treatment, the rats successfully reduced the expression of DNMT1 and MS protein to about 7% of stained cells (**Figure 3C**). This result demonstrates the hypermethylation activity in HCC-developed rats indicated by the DNMT1 and MS expression profiles, which have been reduced upon treatment with GA or a combination of GA and Dox.

### Methylomic changes in NF-kb and STAT-3 in rats with HCC resorted in response to GA treatment

**Figure 5:**
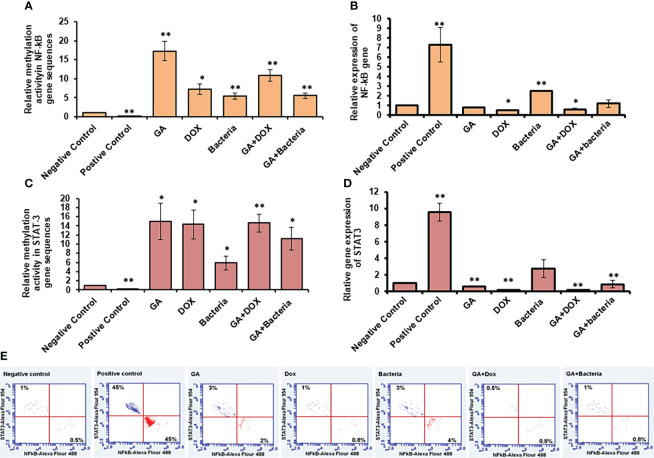
Relative methylation activity of NF-Kb and STAT3 based on RNA expression. **(A, C)** Relative methylation activity NF-kB and STAT-3 gene sequence indicated by fold change that significantly increased upon GA treatment and its combination with DOX or the bacteria. **(B, D)** relative expression of NF-kB and STAT-3 genes significantly reduced upon GA treatment and its indicated combination. Protein expression profile of NF-kB and STAT3 indicated by red and blue dotes using secondary antibodies Alex Flour-488 and 594, respectively **(E)**. *statistically significant difference as compared with the controls (P, 0.05 for each). While, ** statistically significant difference as compared with the controls (P, 0.01 for each).

NF-kb and STAT3 play an essential role in developing and regulating liver cancer. Accordingly, compared to healthy rats, we examined the methylation activity in the coding sequences of NF-kb and STAT-3 in rats with HCC (negative control). We found that the methylation activity in the coding sequence of both NF-kb and STAT3 was significantly reduced in rats with HCC while significantly increasing upon GA treatment and its combination with DOX or the bacteria (**Figures 5A, C**; **Supplementary Table 3**). Notably, the relative gene expression of NF-kb and STAT-3 significantly reduced upon GA treatment, and it indicated a combination, while their expression dramatically increased in rats with HCC (**Figures 5B, D**; **Supplementary Table 4**). These results demonstrate the potential methylomic changes in the coding sequence of NF-kb and STAT3 in rats with HCC and suggest the possible regulatory role of GA in their expression during HCC treatment. Furthermore, the protein expression profile of both NF-kB and STAT3 was markedly increased in rats with HCC (positive control) in almost 45% of stained cells, as indicated by the flow cytometry assay (**Figure 5E**). Meanwhile, their expression profile almost retched the same level of negative control upon treatment with indicated agents, indicating the role of each agent and indicated combination in regulating the expression of oncoprotein, NF-kB, and STAT3 (**Figure 5E**).

### Restoring of TET1 and DLC1 expression profile in rats with HCC in response to GA treatment

**Figure 6:**
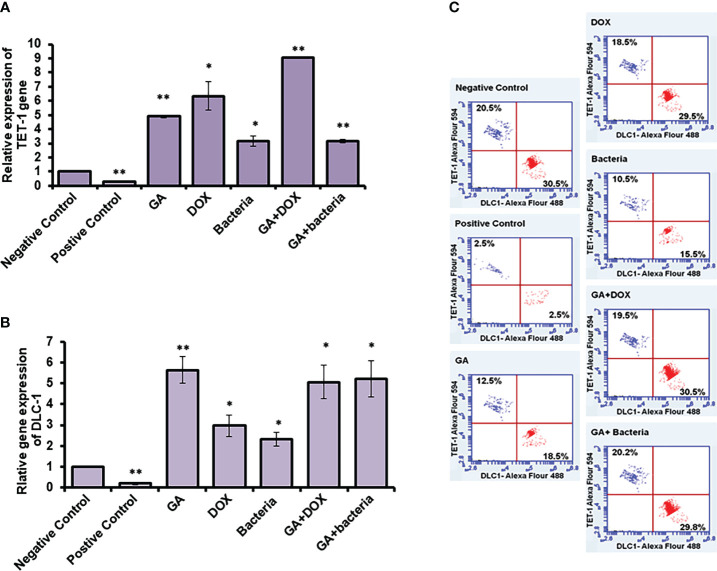
Relative expression of TET-1 and DLC-1 gene expression in GA and combination of GA with bacteria-treated rats. **(A, B)** Relative gene expression of TET-1 and DLC-1 indicated by fold change that was subjected to GA and combination of GA with Bacteria (*lactobacillus rhamanosus*) comparison with control. **(C)** Quantification of protein profile of TET-1 and DLC-1 in treated rats indicated by flow cytometry. *statistically significant difference as compared with the controls (P, 0.05 for each). While, ** statistically significant difference as compared with the controls (P, 0.01 for each).

TET-1 and DLC-1 gene expression was quantified using RT-PCR. The relative gene expression of TET-1 was significantly downregulated in rats with well-differentiated HCC (positive control) (almost 90% inhibition). In contrast, the rats treated with different agents, such as GA, bacteria, a combination of GA with bacteria, and GA with DOX, showed significant upregulation in the TET-1 and DLC-1 genes, as **Figure 6A** and **Supplementary Table 2** displayed. The relative gene expression of DLC-1 was significantly reduced in rats with well-differentiated HCC (approximately 90% inhibition). However, the relative gene expression of DLC-1 was significantly upregulated in rats treated with GA, bacteria, a combination of GA with bacteria, and GA with DOX (**Figure 6C**; **Supplementary Table 2**). Flow cytometric analysis was used to check the kinetic protein expression, as shown in **Figure 6B**. The protein profile of TET-1 in treated rats with GA showed detectable expression in more than 13% of stain cells, while its protein expression was dramatically reduced to less than 3% in positive rats. The protein expression of DLC-1 in rats treated with GA was markedly detected in almost 18.5% of stain cells, whereas less than 3% of stain cells were found in positive rats. Interestingly, the combination of GA with DOX showed potent expression of both TET-1 and DLC-1 in approximately 19.5% and 30% of stained cells, respectively. Likewise, the combination of GA with the bacteria showed competent expression of both TET-1 and DLC-1 in approximately 20.2% and 29.8% of stained cells, respectively. The results demonstrate the activity of GA and its combination with DOX or bacteria to increase the expression of both the TET-1 and DLC-1 genes.

## Discussion

DNA methylation is a hallmark of the epigenetic process because it impacts multigenetic processes like transcription and development. DNA methylation is defined as the conversion of a methyl group onto the C5 position of 5′-CpG-3′ dinucleotides to form 5-methylcytosine (5mC), This process is catalyzed by DNA methyltransferase (DNMT) with S-adenosyl methionine (SAM) as the active methyl donor. The balance between DNA methyltransferases and demethylases is necessary for genomic methylation homeostasis. Imbalances in genomic methylation homeostasis led to different diseases, including cancer (19). In the current study, methylation and demethylation alteration of HCC induced by the chemotoxic model by using diethylnitrosamine (DEN) were used to induce hepatic carcinogenesis and were treated with GA associated with DOX and probiotics. Recently, epigenome-targeted therapy has been a promising strategy in cancer therapy, including signal transduction and tumor suppressor genes (14). Previous articles clarified the hepatoprotective effects of GA, including inhibition of hepatic apoptosis, necrosis, and anti-hepatic fibrosis due to its anti-oxidative activity (8). Interestingly, GA decreases the ROS, which leads to down-regulating the cytotoxicity in hepatocytes induced by bile acid, involving c-Jun N-terminal kinase (JNK) signaling pathways and caspases. Also, GA suppressed caspase-10 by targeting Fas ligand and TRAIL-induced activation, leading to protection of hepatocytes (40). Importantly, GA has anticancer activities against HCC in hepatocellular carcinoma (HepG2) by inducing apoptosis and cell cycle arrest in the G-1 phase. Previous works indicated the plausible mechanism of GA against HCC related to activate caspase-8 and reduction of antiapoptotic proteins (Bcl-2 and Bcl-xL), leading to apoptosis through the downstream mitochondrial pathway (41). Interesting, the oral administration of L. rhamnosus activated the remodeling of the DNA methylation code at the BDNF and Tph1A promoter genes in the gut, which emphasized that the change in composition of the microbiota has a significant effect on the host epigenetic landscape and may lead to a long-term effect on specific gene regions (42). In our work, quantitative real-time PCR was used with the mRNA of liver-treated rats to determine the relative gene expression of TET-1 and DLC-1 at the RNA level; thus, in rats treated with GA, a combination of GA with bacteria and GA with DOX, the relative gene expression of TET-1 at the RNA level was significantly upregulated. In rats treated with GA, a combination of GA and bacteria, and a combination of GA with DOX, the relative gene expression of DLC-1 was significantly upregulated. The expression of both TET-1 and DLC-1 proteins in the liver tissue of treated rats was assessed using flow cytometry. Furthermore, our findings suggest that the treatment with GA and its combination with DOX or bacteria significantly increased the expression of both TET-1 and DLC-1 proteins. To investigate the possibility of epigenetic changes in the coding sequences of the TET-1 and DLC-1 genes, qRT-PCR was used to amplify the digested genes with methylation-dependent and methylation-sensitive enzymes (39). Meanwhile, GA treatment reduced methylation activity in both TET-1 and DLC-1. Furthermore, combining GA with DOX or bacteria effectively avoids methylation activity in both genes. It is well known that STAT3 contributes to the development of HCC by promoting growth, anti-apoptosis, migration, invasion, angiogenesis, cancer stem cell characteristics, and immune suppression of cancer cells. These actions are typically carried out by modulating the transcription of several oncogenic target genes (14). After being released from an inhibitor of NF-kB (IkB), NF-kB translocates from the cytoplasm to the nucleus in response to proinflammatory stimuli, where it is phosphorylated by IkB and degraded. A panel of cytokines, including IL-6, is secreted in greater amounts at the sites of inflammation when NF-kB is activated (43). NF-kb and STAT3 play an important role in the development and regulation of liver cancer, so we used the complementary DNA (cDNA) prepared from total RNA to examine the methylation activity in the coding sequences of NF-kb and STAT3 in rats with HCC compared to the negative control. Accordingly, we found that the potential methylomic alterations in the STAT3 and NF-kb coding sequences in rats with HCC and the possible regulation of GA in their expression during treatment for HCC are suggested. Likewise, by blocking SIRT3, GA prevented colorectal cancer cells from proliferating, migrating, or invading other tissues. As a result, GA may one day be used to treat colorectal cancer (44). GA has an anti-HCC effect and a great liver targeting ability, which suggests that the GA-modified novel delivery systems have good pharmacokinetics and could be a promising strategy for using GA in HCC therapy. Based on the results, methylation activity and epigenetic-targeted therapy are promising anticancer treatment strategies.

## Conclusion

In this work, we investigated the activity of GA in combination with different agents to reduce the methylation activity of HCC and improve the tissue texture of the HCC tissue by using multi-techniques such as flow cytometry and RT-PCR. The results displayed the activity of GA to reduce the methylation activity in TET-1 and DLC-1 by 33.6% and 78%, respectively. As compared with the positive control. Furthermore, the association of GA with DOX avoided the methylation activity by 88% and 91% for TET-1 and DLC-1, respectively, as compared with the positive control. Similarly, the combined use of GA with probiotics suppressed the methylation activity in the TET-1 and DLC-1 genes by 75% and 81% for TET-1 and DLC-1, respectively. Also, GA and its combination with bacteria attenuated the adverse effect in hepatocarcinogenesis rats by altering potential methylomic genes such as NF-kb and STAT3 genes by 76% and 83%, respectively. Finally, we hypothesize that GA treatment affects TET-1 and DLC-1 gene expression by decreasing the methylation activity in promoting and coding sequences.

## Data availability statement

The original contributions presented in the study are included in the article/**Supplementary Material**. Further inquiries can be directed to the corresponding author.

## Ethics statement

All animal-handling actions as well as samples gathering and discarding will be conferring to the regulations of the Institutional Animal Care and Use Committee (IACUC) with oversight of the faculty of Veterinary Medicine, University of Sadat City, Sadat City, Egypt (Ethical approval number: VUSC-025-1-22). The studies were conducted in accordance with the local legislation and institutional requirements. Written informed consent was obtained from the owners for the participation of their animals in this study.

## Author contributions

Conceptualization: AAE, HK, and HM; Formal analysis: AAE and HK; funding: AAE; Investigation: AN, AAE, HK, and DMoh; Methodology: AAE and HK: Project administration: AAE; Resource: II, PR, and FA-S; Supervision: AAE and HK; Validation: AAE, HK, and HM; Visualization: AH, AAE, and HK; Writing original draft: AH, AAE, HK, and II. Writing -review edition: AH; Editing: AH, HK, and PR. All authors contributed to the article and approved the submitted version.

## Supplementary material

Supplementary Figure 1 Chemical induction of HCC in albino rats treated with different effectors.
