# Supplementary figures and images for: Amelioration effect of 18β-Glycyrrhetinic acid on methylation inhibitors in hepatocarcinogenesis -induced by diethylnitrosamine
Source: Front Immunol. 2024 Jan 15;14:1206990. doi: 10.3389/fimmu.2023.1206990 (PMC10844948; doi:10.3389/fimmu.2023.1206990)

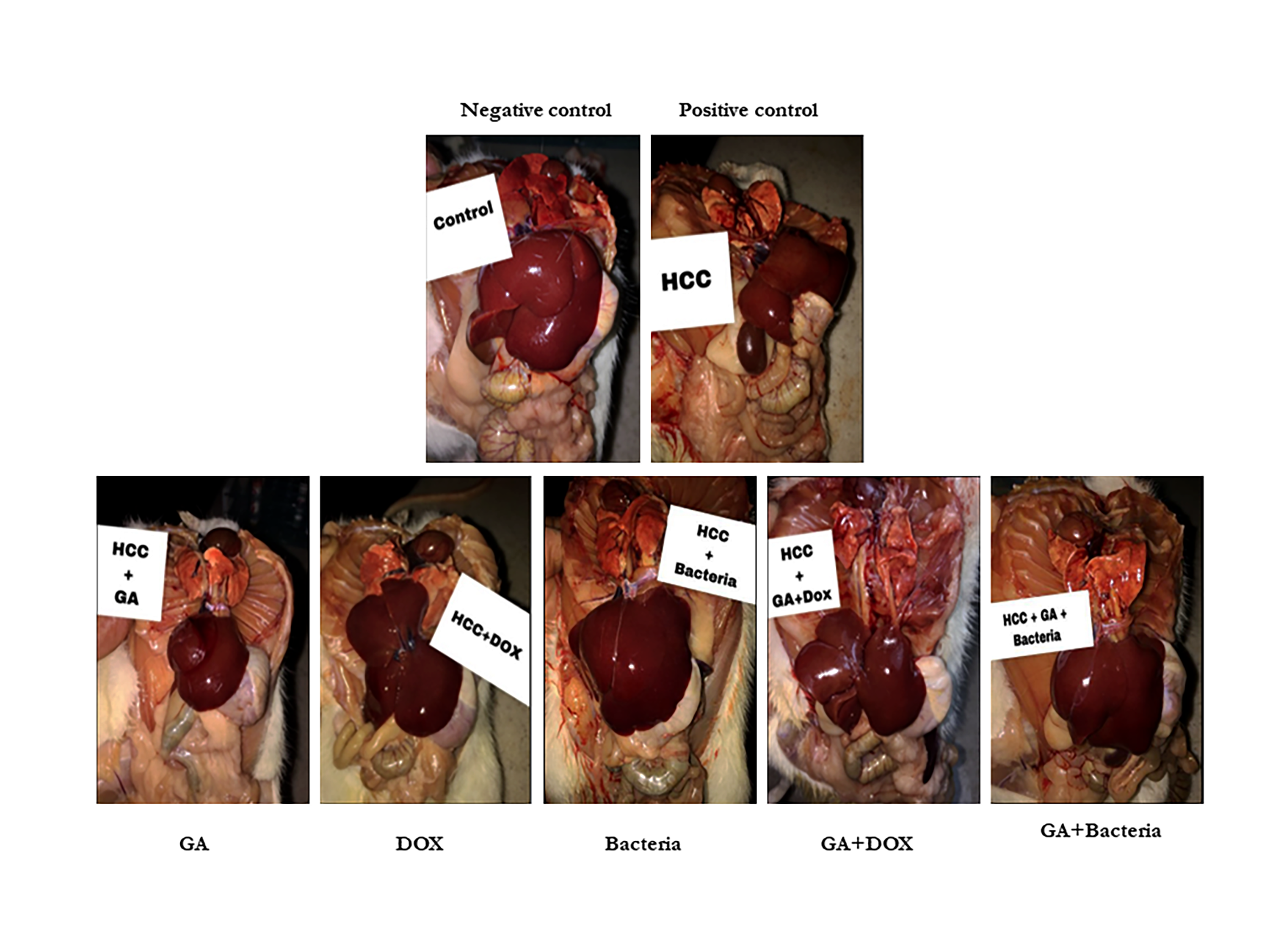

Supplement: Supplementary Figure 1 — Chemical induction of HCC in albino rats treated with different effectors. [file Image_1.tif]
