# Supplementary material for: Amelioration effect of 18β-Glycyrrhetinic acid on methylation inhibitors in hepatocarcinogenesis -induced by diethylnitrosamine
Source: Front Immunol. 2024 Jan 15;14:1206990. doi: 10.3389/fimmu.2023.1206990 (PMC10844948; doi:10.3389/fimmu.2023.1206990)
Supplement: Supplementary file 2 [file DataSheet_1.pdf]

S.Table 1: Relative methylation activity in *TET-1* and *DLC-1* gene sequence

These findings suggest that GA, in addition to DOX, has anti-methylation properties during HCC treatment.

genes	conditions	expression fold changes	standard deviation	student t-tails	P-values
**TET-1**	**Negative Control**	1.0000	0		
	**Postive Control**	16.9159**	0.4974	0.000488291	**< 0.01**
	**GA**	10.8601*	2.2720	0.025540444	**< 0.05**
	**DOX**	2.6889	0.7921	0.094641067	**< 0.05**
	**Bacteria**	4.3985	1.2958	0.065633735	**< 0.05**
	**GA+DOX**	2.1534	1.5813	0.410790542	**< 0.05**
	**GA+bacteria**	2.6737	1.9151	0.341822463	**< 0.05**
**DLC-1**	**Negative Control**	1.000	0		
	**Postive Control**	54.2336**	3.5855	0.001493261	**< 0.01**
	**GA**	10.5813*	1.8778	0.018667712	**<0.05**
	**DOX**	7.1886	0.3173	0.001311764	
	**Bacteria**	42.7993**	8.5432	0.020255842	**<0.01**
	**GA+DOX**	2.7498	1.5754	0.256845361	**<0.01**
	**GA+bacteria**	9.3147*	1.8593	0.024102292	**<0.05**

S.Table 2: Relative expression of DLC-1 and TET-1

Relative gene expression of TET-1 and DLC-1 indicated by fold change that was subjected to GA and combination of GA with Bacteria (*lactopacillus rhamanosus*) comparison with control.

genes	conditions	expression fold changes	standard deviation	student t-tails	P-values
**DLC-1**	**Negative Control**	1	0		
	**Postive Control**	0.192**	0.080	0.00488	< **0.01**
	**GA**	5.645**	0.650	0.00966	< **0.01**
	**DOX**	2.973*	0.523	0.03342	< **0.05**
	**Bacteria**	2.312*	0.335	0.03115	< **0.05**
	**GA+DOX**	5.064*	0.806	0.01909	< **0.05**
	**GA+bacteria**	5.219*	0.850	0.01972	< **0.05**
**TET-1**	**Negative Control**	1.000	0		
	**Postive Control**	0.290**	0.023	0.00052	< **0.01**
	**GA**	4.893**	0.098	0.00032	< **0.01**
	**DOX**	6.340*	1.021	0.01779	< **0.05**
	**Bacteria**	3.142*	0.360	0.01380	< **0.05**
	**GA+DOX**	9.069**	0.027	0.00030	< **0.01**
	**GA+bacteria**	3.158**	0.119	0.00394	< **0.01**

S.table 3: Relative methylation activity of NF-kB and STAT-3 gene sequence

These data indicate by fold change that both NF-kb and STAT3 significantly reduced in rats with HCC, while significantly increased upon GA treatment and its combination with DOX or the bacteria

genes	conditions	expression fold changes	standard deviation	student t-tails	P-values
**NF-kB**	**Negative Control**	1.0000	0.0000		
	**Postive Control**	0.2665**	0.0898	0.00740	< **0.01**
	**GA**	17.2411**	2.5260	0.01188	< **0.01**
	**DOX**	7.2794*	1.4178	0.02455	< **0.05**
	**Bacteria**	5.4937**	0.8049	0.01565	< **0.01**
	**GA+DOX**	10.8362**	1.5876	0.01277	< **0.01**
	**GA+bacteria**	5.5629 **	0.7070	0.01179	< **0.01**
**STAT-3**	**Negative Control**	1.000	0		
	**Postive Control**	0.1963**	0.0096	0.00007	< **0.01**
	**GA**	14.9394*	3.9792	0.03841	< **0.05**
	**DOX**	14.3450*	3.1383	0.02655	< **0.05**
	**Bacteria**	5.8606*	1.5610	0.04789	< **0.05**
	**GA+DOX**	14.6346**	1.9310	0.00988	< **0.01**
	**GA+bacteria**	11.2548*	2.4624	0.02764	< **0.05**

S.table 4: Relative genes expression of both NF-Kb and STAT-3

Relative gene expression of NF-kb and STAT3 significantly reduced upon GA treatment and its indicated combination, while their expression dramatically increased in rats with HCC

genes	conditions	expression fold changes	standard deviation	student t-tails	P-values
**NF-kB**	**Negative Control**	1.0000	0.0000		
	**Postive Control**	7.319**	1.776	0.03730	< **0.01**
	**GA**	0.803**	0.087	0.08547	< **0.01**
	**DOX**	0.538*	0.082	0.01531	< **0.05**
	**Bacteria**	2.538**	0.058	0.00072	< **0.01**
	**GA+DOX**	0.613*	0.104	0.03449	< **0.05**
	**GA+bacteria**	1.215**	0.412	0.53779	< **0.01**
**STAT-3**	**Negative Control**	1.000	0		
	**Postive Control**	9.557**	1.049	0.00743	< **0.01**
	**GA**	0.612**	0.031	0.00312	< **0.01**
	**DOX**	0.176**	0.009	0.00006	< **0.01**
	**Bacteria**	2.744*	1.078	0.14932	< **0.05**
	**GA+DOX**	0.213**	0.057	0.00265	< **0.01**
	**GA+bacteria**	0.898**	0.449	0.77889	< **0.01**
